# Spontaneous dissociation of Co_2_(CO)_8_ and autocatalytic growth of Co on SiO_2_: A combined experimental and theoretical investigation

**DOI:** 10.3762/bjnano.3.63

**Published:** 2012-07-25

**Authors:** Kaliappan Muthukumar, Harald O Jeschke, Roser Valentí, Evgeniya Begun, Johannes Schwenk, Fabrizio Porrati, Michael Huth

**Affiliations:** 1 Institut für Theoretische Physik, Goethe-Universität, Max-von-Laue-Straße 1, 60438 Frankfurt am Main, Germany; 2 Physikalisches Institut, Goethe-Universität, Max-von-Laue-Straße 1, 60438 Frankfurt am Main, Germany; 3 present address: Empa, CH-8600 Dübendorf, Switzerland

**Keywords:** Co_2_(CO)_8_, deposition, dissociation, EBID, FEBID, precursor, radiation-induced nanostructures

## Abstract

We present experimental results and theoretical simulations of the adsorption behavior of the metal–organic precursor Co_2_(CO)_8_ on SiO_2_ surfaces after application of two different pretreatment steps, namely by air plasma cleaning or a focused electron beam pre-irradiation. We observe a spontaneous dissociation of the precursor molecules as well as autodeposition of cobalt on the pretreated SiO_2_ surfaces. We also find that the differences in metal content and relative stability of these deposits depend on the pretreatment conditions of the substrate. Transport measurements of these deposits are also presented. We are led to assume that the degree of passivation of the SiO_2_ surface by hydroxyl groups is an important controlling factor in the dissociation process. Our calculations of various slab settings, using dispersion-corrected density functional theory, support this assumption. We observe physisorption of the precursor molecule on a fully hydroxylated SiO_2_ surface (untreated surface) and chemisorption on a partially hydroxylated SiO_2_ surface (pretreated surface) with a spontaneous dissociation of the precursor molecule. In view of these calculations, we discuss the origin of this dissociation and the subsequent autocatalysis.

## Introduction

In recent years, focused electron beam induced deposition (FEBID) has emerged as a versatile, high-resolution technique for nanostructure fabrication in contrast to the more conventional nanolithographic techniques. In FEBID, a previously adsorbed precursor gas is dissociated in the focus of an electron beam. The nonvolatile part of the dissociation products remains as a deposit whose shape and position can be accurately controlled by the lateral positioning of the electron beam in an electron microscope [1–5]. Mostly gaseous, e.g., W(CO)_6_, Fe(CO)_5_, and CH_3_C_5_H_5_Pt(CH_3_)_3_ [6–9], but also liquid organometallic precursors (chloroplatinic acid) [10] are used to deposit metals or metal composites on selected regions of the substrates. Deposits with a wide spectrum of properties and composition can be consequently obtained due to the availability of suitable precursors [1–2]. Co_2_(CO)_8_ has been recently used as a precursor molecule in FEBID to obtain granular deposits with differing compositions of cobalt [11]. Electronic and physical properties, such as grain size and metal content of these deposits, depend strongly on the deposition and pretreatment conditions of the substrate. By regulating these conditions, deposits of desired size and different Co content can be fabricated [12–15]. For example, granular Co-nanostructures suitable for micro Hall sensing devices [16] were thus obtained. Very recently this precursor has also been used in combination with the precursor CH_3_C_5_H_5_Pt(CH_3_)_3_ to fabricate nanogranular CoPt-C structures with CoPt nanocrystallites having the L1_0_ crystal structure with hard-magnetic properties [17]. Also, it has been shown that, under well-controlled conditions, Co line structures with a width down to 30 nm are feasible [18–19]. These findings make FEBID with the Co-precursor particularly attractive for the fabrication of micromagnetic structures in the sub-100 nm regime, relevant for studies of the domain wall dynamics [20], the Barkhausen effect in single-domain-wall structures [21] and dipolar coupling effects [22]. While several experimental studies based on infrared spectroscopy [23–26] and theoretical [27–30] studies on Co_2_(CO)_8_ are available in the literature, an issue that remains unclear so far is the possible tendency of this precursor to spontaneously dissociate on SiO_2_ surfaces, as well as to autocatalytically grow by spontaneous decomposition on existing Co clusters. Similar features have been reported to be exhibited by Fe(CO)_5_ [31–32]. In order to evaluate the previous effects in the FEBID process, it is mandatory to acquire an in-depth knowledge of the interactions between the precursor molecule Co_2_(CO)_8_ and SiO_2_ surfaces, representing the different pretreatment conditions of the substrate [33].

In the present work, we report on experimental results of Co deposition by spontaneous dissociation of the precursor Co_2_(CO)_8_ on untreated and two differently pretreated SiO_2_ surfaces (by an air plasma cleaning process and a pregrowth electron irradiation of selected areas). To our knowledge, no systematic theoretical studies with in-depth DFT calculations on Co_2_(CO)_8_ adsorbed on different SiO_2_ surfaces are available. Therefore, we extent the study using density functional theory (DFT) calculations on slabs representing the various SiO_2_ surface conditions, and we aim to relate the observations to the plasma and electron irradiation conditions prevailing in FEBID experiments.

## Experimental

Cobalt growth and imaging experiments were carried out at room temperature in a dual-beam scanning electron microscope (FEI Nova NanoLab 600) with a Schottky electron emitter. A plasma source using ambient air at a chamber pressure of 1 × 10^−4^ to 5 × 10^−4^ mbar was used for the surface-activation experiment (GV10x Downstream Asher, ibss Group). Electron pregrowth irradiation experiments were carried out at 5 kV beam voltage and 1.6 nA beam current. Si(100) (p-doped) substrates with thermal oxide layers of 50 nm up to 285 nm were used. Before use, the substrates were chemically cleaned by acetone, isopropanol and distilled water in an ultrasound bath. In the plasma activation experiments the silica sample surface (285 nm oxide layer) was exposed to the plasma discharge for 75 min after the scanning electron microscope (SEM) chamber had been evacuated to its base pressure of about 5 × 10^−6^ mbar. After the plasma treatment the chamber was again evacuated to base pressure and Co-precursor flux was admitted to the chamber by opening the valve of a home-made gas injection system for 30 min, causing a pressure increase to 3 × 10^−5^ mbar, which dropped within ten minutes to about 6 × 10^−6^ mbar. The gas injection system employs a stainless-steel precursor capsule with a fine-dosage valve. The precursor temperature was set by the ambient conditions to 27 °C. From the known precursor temperature and associated vapor pressure, as well as the geometry of our gas injection system we can roughly estimate the maximum molecular flux at the substrate surface to be 1.4 × 10^17^ cm^−2^ s^−1^ following [34].

In the second series of experiments the untreated silica surface was pregrowth irradiated with a focused electron beam, which was moved in a raster fashion (dwell time 100 μs, pitch 20 nm) for 30 min over a rectangular region of 3.7 × 1.0 μm^2^ bridging the gap between two prepatterned Cr/Au electrodes. The background pressure during the irradiation process was 6 × 10^−6^ mbar. Within the 30 min irradiation time about two thousand passes of the rectangular pattern were performed, amounting to an overall dose of 0.78 μC/μm^2^. After this treatment the Co-precursor was admitted to the SEM chamber and the current between the electrodes was measured at a fixed bias voltage of 10 mV as a function of time (see below in Figure 3b). By this method the formation of a conducting path between the metallic electrode can be conveniently followed and gives a first indication of the spontaneous formation of a deposit. After about 20 min the injection was stopped, and the SEM chamber was flushed with dry nitrogen and evacuated again for image acquisition.

## Computational details

We performed spin-polarized density functional theory (DFT) calculations within the generalized gradient approximation in the parametrization of Perdew, Burke and Ernzerhof (PBE) [35–36]. Corrections for long-range van der Waals interactions [37–38] were included in all calculations. We worked with a kinetic energy cut-off of 400 eV and relaxed all the ions with the conjugate gradient scheme until the forces were less than 0.01 eV/Å. In order to reproduce the experimental settings, untreated SiO_2_ surfaces were described in terms of fully hydroxylated substrates, while pretreated SiO_2_ surfaces were described in terms of partially hydroxylated substrates [39–41]. Our (fully and partially hydroxylated) SiO_2_ substrates consist of four layers of (3 × 3) supercells of β-cristobalite primitive unit cells. We calculated total energy differences Δ*E* for substrates, precursor molecules, and the complex of the substrate with adsorbed precursor molecules, as reported previously [9,33] using the projector augmented wave method [42–43] as implemented in the Vienna Ab-initio Simulation Package (VASP) [44–46]. In the geometry optimizations for the molecule and the substrate models the Brillouin zone was sampled at the Γ point only. In addition, to analyze the molecular orbitals, we employed Turbomole 6.0 [47–48] to optimize the Co_2_(CO)_8_ molecule with triple-zeta valence plus polarization basis sets with the PBE functional using the resolution-of-the-identity (RI) approximation. The Bader charge partition analysis was performed by using the code of Henkelman et al. to determine the charges of individual atoms [49–50].

## Results and Discussion

### Formation of Co from Co_2_(CO)_8_ on pretreated SiO_2_ surfaces

**Figure 1:**
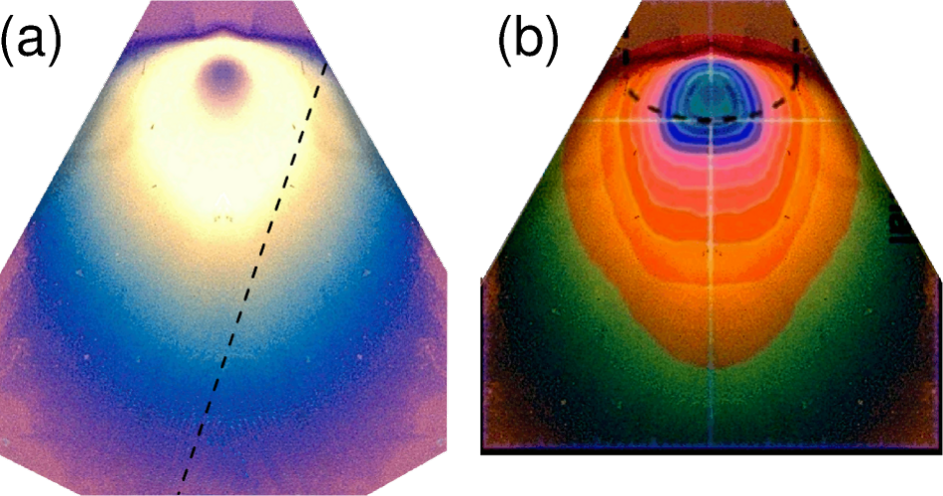
(a) Optical micrograph of the Co dissociation product on the plasma-activated silica surface. The deposit mimics the flux profile set by the gas injection needle. The dashed line represents the rightmost substrate edge. The deposit profile to the right of the dashed line was complemented by image processing from the left side for ease of comparison. (b) Overlay of the calculated precursor flux profile from [34] (contour lines) with the isotropically scaled optical microsocope image of the deposit profile shown in (a).

In Figure 1a we present an optical micrograph of a spontaneous dissociation product obtained on the plasma pretreated SiO_2_ surface. A Co-rich layer of varying thickness has been formed, whose lateral shape clearly depicts the precursor flux profile imposed by the gas injection needle. This profile appears in Figure 1b and is in excellent agreement with simulations of the precursor flux presented in [34]. It should be stressed that no such spontaneous growth was observed on the untreated SiO_2_ surface after 30 min exposure to the Co-precursor. Only for extended exposure times (30 min or longer) do we find evidence of the tendency for spontaneous dissociation also on the untreated surfaces. At this stage we are led to assume that the untreated SiO_2_ surface, usually hydroxylated after chemical cleaning as performed by us, shows a weak tendency to induce spontaneous dissociation of the Co-precursor. Partial or full removal of the hydroxyl surface passivation layer leads to an increased driving force for dissociation. This will be discussed in more detail in the the next section in which we present results obtained in the framework of DFT calculations concerning the adsorption behavior and stability of the Co-precursor on the SiO_2_ surface under different hydroxylation conditions.

**Figure 2:**
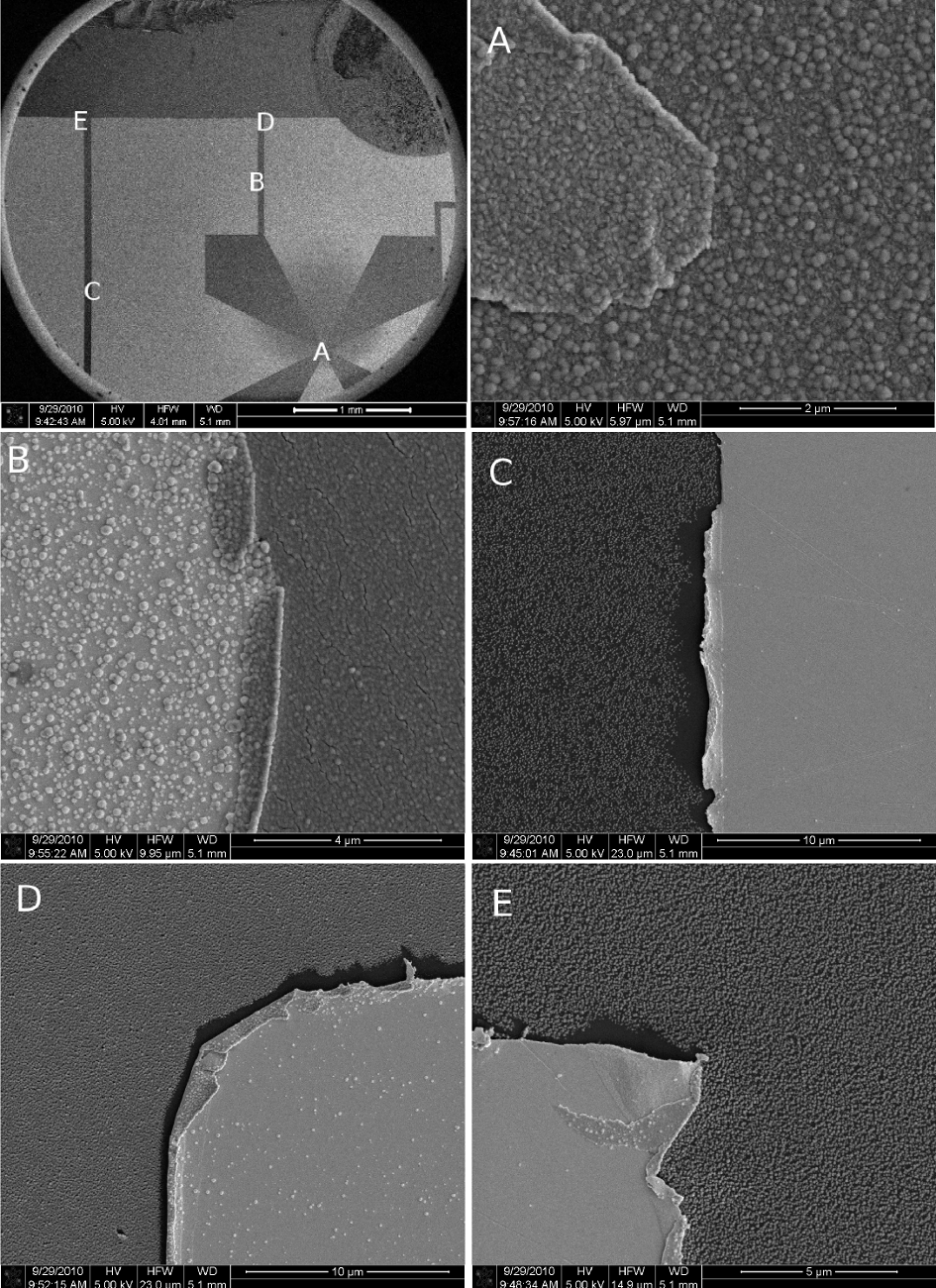
SEM images of Co deposited on the plasma-pretreated silicon oxide and gold. The picture on the top left is an overview of a SiO_2_ surface prepatterned with Cr/Au contact structures. The labeling A–E indicates regions of different precursor flux, which was centered at A. The gas injection capillary is visible on the upper right. Gold surfaces appear as bright regions, SiO_2_ surfaces as dark regions. Selected area SEM images are represented in images A–E.

In a follow-up experiment, we analyzed the influence of a metallic surface, as provided by Cr/Au (20 nm/80 nm) contact structures, on this spontaneous dissociation process (see Figure 2). Inspection of the surface at various positions on the SiO_2_ surface and the Au/Cr contact structures, and after 30 min plasma treatment and 10 min precursor flux exposure reveals clear differences. In regions of maximum precursor flux (see position A in Figure 2) we observe slight differences in the morphology of the formed Co clusters on the electrodes as compared to the growth on the SiO_2_ surface. In particular, a reduced average Co grain size and grain density on the Au electrodes is observed. In regions of low precursor flux, only small islands of the dissociation product are visible on the Au contacts, whereas the SiO_2_ surface is mostly covered (see region D and E in Figure 2). Evidently, the surface state of the plasma-pretreated SiO_2_ surface provides a stronger driving force for the spontaneous precursor dissociation.

**Figure 3:**
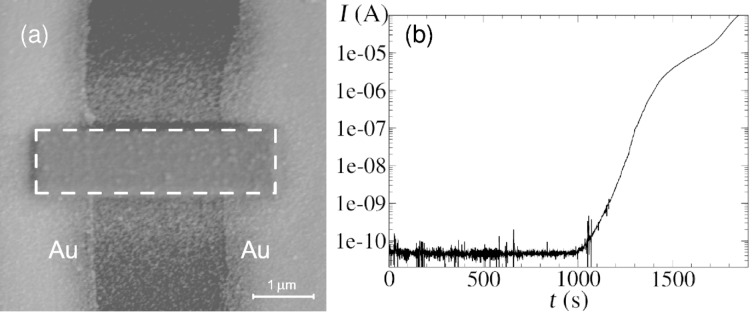
(a) SEM micrograph of Co deposit formed after electron pre-irradiation of the rectangular area depicted by the dashed contour. (b) Time-dependence of the current flow between the Au electrodes at fixed bias voltage (10 mV) as the Co deposit forms spontaneously. The current increase after closing the valve of the gas injector (1200 s) indicates that residual precursor molecules in the SEM vacuum chamber are continuously dissociated resulting in a further increase of the thickness of the Co layer. After exposure of the sample to air the layer thickness was determined by atomic force microscopy and found to be approximately 50 nm.

We now turn to the results obtained on the SiO_2_ surface with selected regions that were pretreated by electron irradiation. In Figure 3a we show the SEM micrograph of a Co-containing deposit obtained in a region in which the electron beam was rastered over a rectangular area of the SiO_2_ surface for 10 min before admission of the precursor for 20 min. As is evident from the figure, a deposit between the Au electrodes was formed, whose outline represents a slightly blurry replica of the previously activated region. According to our Monte Carlo simulations using CASINO V2.42 [51] the extent of the blurred region corresponds roughly to the range of the backscattered electrons. Additional islands of the spontaneous dissociation products are visible away from the pretreated region. The density of these islands drops off to zero over a length scale of about 1 μm.

**Figure 4:**
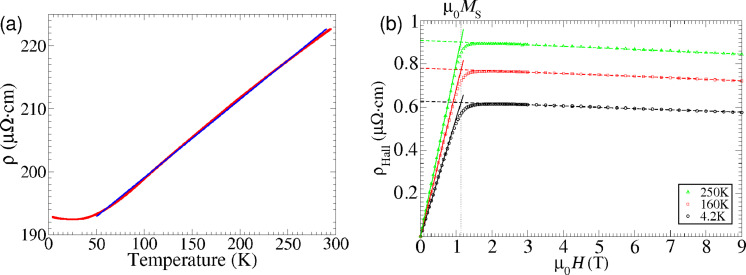
(a) Temperature dependence of resistivity of Co deposit grown on the plasma-activated SiO_2_ surfaces. The lateral shape of the deposit for resistivity and Hall effect measurements was defined by a lift-off procedure of a photolithographically defined resist pattern on which the plasma-activated growth had been performed. The deposit height was determined as 55 nm by atomic force microscopy. Blue line: linear fit between 50 and 290 K. (b) Hall resistivity as function of magnetic field, measured at different temperatures as indicated. The saturation magnetization is denoted as μ_0_*M*_S_.

An energy dispersive X-ray (EDX) analysis of the dissociation products obtained by the plasma activation and pregrowth electron irradiation treatment reveals a Co content of approximately 95 and 76%, respectively. In subsequent resistivity measurements we found a room temperature resistivity of 223 and 480 μΩ·cm, respectively. This is about a factor of 5 larger than the room temperature resistivity found for FEBID-grown Co nanowires employing the same precursor [52–53]. A larger degree of grain boundary scattering in the spontaneously formed deposit, as well as a possibly higher carbon content may be the cause for this enhanced resistivity. We also performed temperature-dependent resistivity measurements (Figure 4a) as well as Hall effect measurements (Figure 4b) for the sample grown on the plasma-activated silica. The samples grown under pre-irradiation conditions are unstable under thermal stress and could not be measured below room temperature. The temperature-dependent resistivity shows a typical metallic behavior as expected for a dirty metal. From the Hall measurement we deduced the saturation magnetization, as indicated in Figure 4b, following established procedures, as detailed in [54].

### Structure and bonding of Co_2_(CO)_8_ on SiO_2_ surfaces

#### Structure of the Co_2_(CO)_8_ molecule

**Figure 5:**
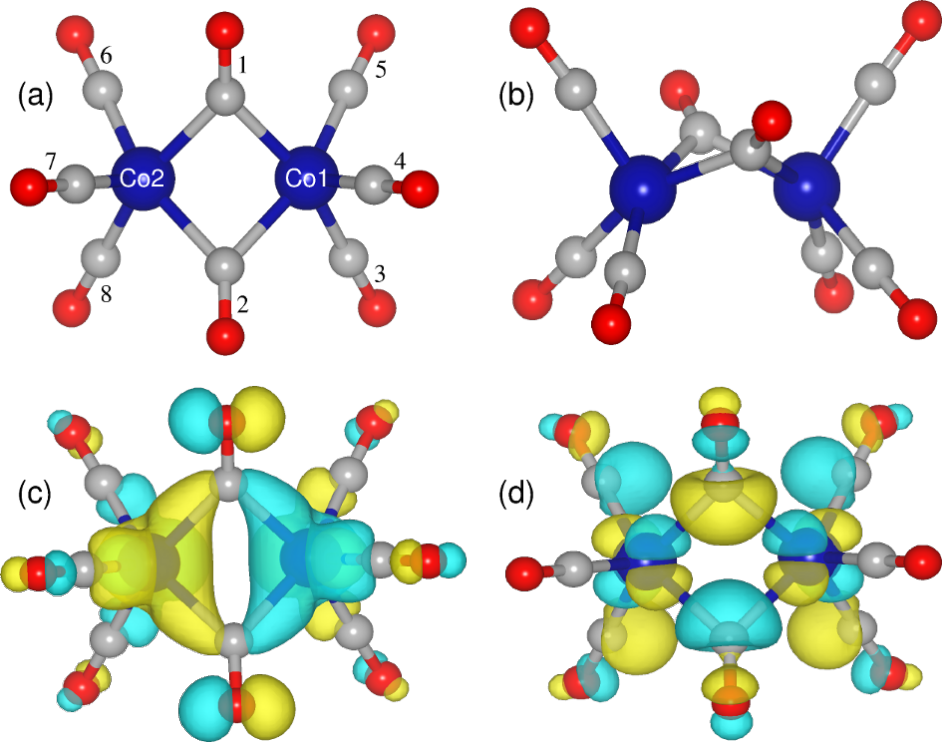
(a) Top and (b) side view of DFT optimized structure of Co_2_(CO)_8_ and its frontier orbitals (c) HOMO and (d) LUMO. Blue, red and grey spheres represent cobalt, oxygen and carbon atoms respectively.

The structure of Co_2_(CO)_8_ has been well studied and found to have a distorted Fe_2_(CO)_9_ structure with one bridge carbonyl less. Sumner et al. reported a *C_s_* symmetric structure resembling the *C*_2_*_v_* symmetry (Figure 5a), which was analyzed by DFT calculations [27]. Less stable *D*_2_*_d_* and *D*_3_*_d_* isomers that do not have the bridging ligands have also been observed in solution [55–57]. The structural parameters obtained from our DFT studies, such as the distance between the two cobalt atoms (2.52 Å) and the distance to the bridging (1.95 Å) and terminal ligands (1.81 Å) from the metal atom, match the reported values well [58]. Further, we find the *D*_3_*_d_* symmetric structure to be less stable by 6.9 kcal/mol with respect to the *C*_2_*_v_* isomer compared to the reported value of 5.8 kcal/mol [27]. Electronic-structure analysis indicates that the highest occupied orbital (HOMO) is dominated by Co 3d orbitals (Figure 5c), and the lowest unoccupied orbital (LUMO) has a significant contribution from the 2p orbitals (Figure 5d) of the carbonyls.

#### Bonding of Co_2_(CO)_8_ molecules on SiO_2_ surfaces

**Figure 6:**
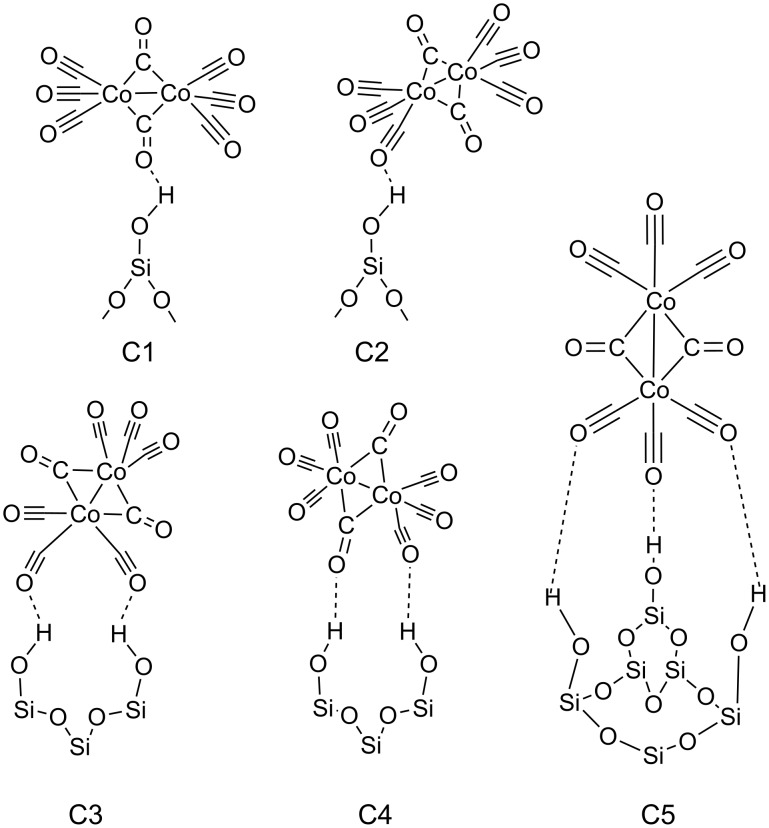
Schematic representation of the starting configurations with possible Co_2_(CO)_8_ orientations, considered in this study, on FOH-SiO_2_ surfaces. In POH-SiO_2_ surfaces some of the OH groups are partially removed in order to simulate the pretreated surfaces.

In general, the interaction of metal carbonyls with hydroxylated oxidic surfaces occurs through the coordination of the basic oxygen of the metal carbonyls with the weakly acidic surface hydroxyls. In this study, we consider fully (FOH) and partially hydroxylated (POH) SiO_2_ surfaces that directly represent the untreated and pretreated surfaces. For the POH-SiO_2_ surfaces three different cases that differ in the degree of hydroxylation, corresponding to an OH vacancy concentration of 11, 22 and 33%, were considered depending upon the orientation of Co_2_(CO)_8_ on the surface [33]. In order to have the most stable bonding configuration of Co_2_(CO)_8_ on these FOH-SiO_2_ and POH-SiO_2_ surfaces, five different orientations (C1 to C5 as shown in Figure 6) were considered. These orientations take into account the possible ways in which the precursor molecule can adsorb on the surface.

**Figure 7:**
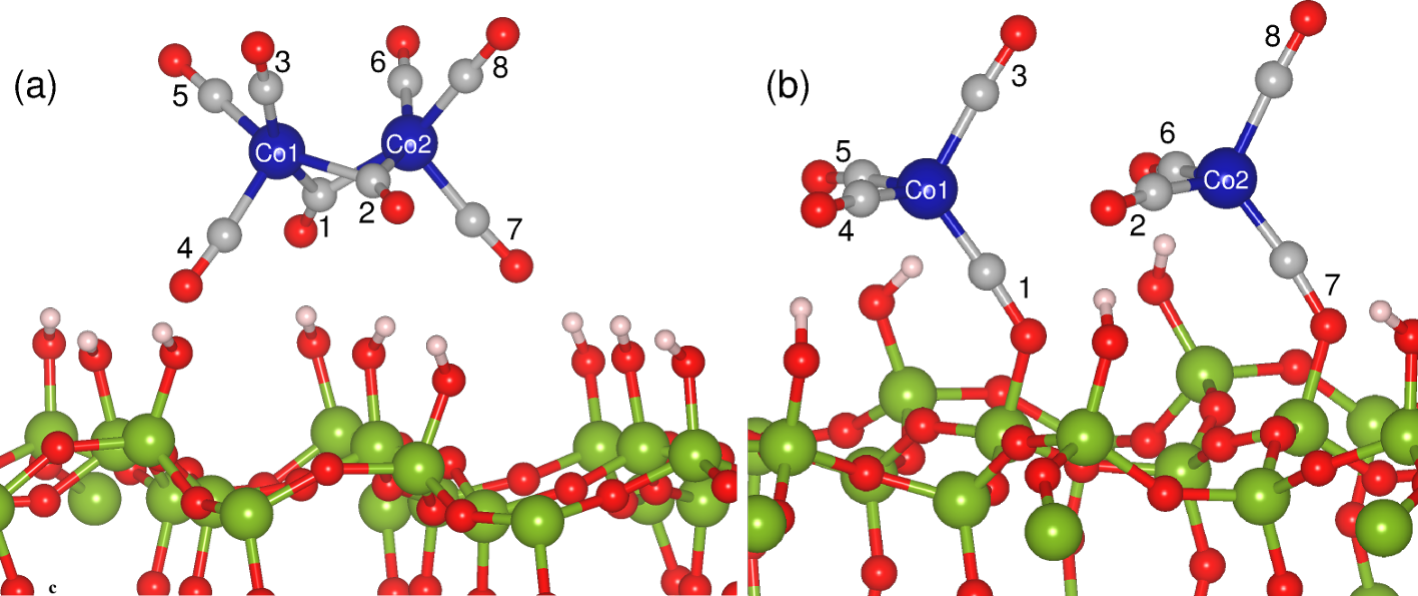
(a) Most stable structure of Co_2_(CO)_8_ on the (a) FOH-SiO_2_ and (b) POH-SiO_2_ surfaces. The molecule dissociates on the POH surfaces into two Co(CO)_4_ ions bonding to a terminal Si of the surface. Green, blue, red and grey spheres represent silicon, cobalt, oxygen and carbon atoms, respectively.

The calculated adsorption energies for the different configurations of Co_2_(CO)_8_ on FOH-SiO_2_ surfaces range from −0.26 to −0.76 eV (Table 1) illustrating that the precursor molecule binds weakly on these surfaces. Bonding through one of the basic bridging ligands (C1) is preferred compared to bonding with one of the terminal ligands of the molecule (C2). However, an interesting result was obtained when relaxations were started with the C4 configuration, in which case the molecule rearranges in such a way that two of its bridging and terminal ligands are oriented towards the surface (Figure 7a), with distances to the surface of 2.08–2.39 Å. The obtained distances agree well with the recently reported hydrogen-bonding distance of tungsten carbonyls with the SiO_2_ substrate [33]. This configuration turns out to be the most stable configuration. The difference in adsorption energy between the C4 configuration and the rest of the configurations ranges between 0.3–0.5 eV. These differences may be small under typical FEBID conditions, in particular if local beam heating has to be taken into account. In this case the molecule is expected to possess random orientations on the fully hydroxylated surface. For the pretreated SiO_2_ surfaces a preferential precursor orientation is expected. It was suggested that the weak interaction between the metal carbonyls and the surface OH groups weakens bonding in the molecule [59]. This is not supported by our calculations, which show negligible changes in the Co–Co and Co–CO bonds of the precursor Co_2_(CO)_8_ on the order of 0.01–0.02 Å.

**Table 1:** Calculated adsorption energies (in eV) of Co_2_(CO)_8_ on SiO_2_ surfaces. Configurations marked with an asterisk change as a result of geometry optimization and are discussed in the text.

configuration	FOH-SiO_2_	POH-SiO_2_

C1	−0.34	−1.69
C2	−0.26	−0.78
C3	−0.47	−2.46^*^
C4	−0.76^*^	−3.54^*^
C5	−0.36	−1.12

In the case of POH-SiO_2_ surfaces, adsorption energies are on the order of −0.78 to −3.54 eV indicating that the molecule is bound strongly to these surfaces. The least stable configuration is C2, in which one of the terminal ligands is bonded to the surface Si atoms. The most stable case, with an adsorption energy of −3.54 eV, is obtained when relaxations are started with C4, in which one bridging and one terminal ligand are involved in bonding to the surface. The most interesting observation in this case, is that the Co_2_(CO)_8_ dissociates spontaneously into two Co(CO)_4_ molecules during geometry optimization (see Figure 7b). This dissociation has also been observed when the molecule interacts with the POH-SiO_2_ surface with two terminal ligands (C3), and has not been observed when the molecule binds either with one bridging or one terminal oxygen (C1, C2). Although one may expect a fragmentation of a Co–C bond to be similar to the W–C bond breaking in W(CO)_6_ [33], the dissociation of Co_2_(CO)_8_ occurs by breaking of the Co–Co bonds. We will discuss this process in the next section.

**Figure 8:**
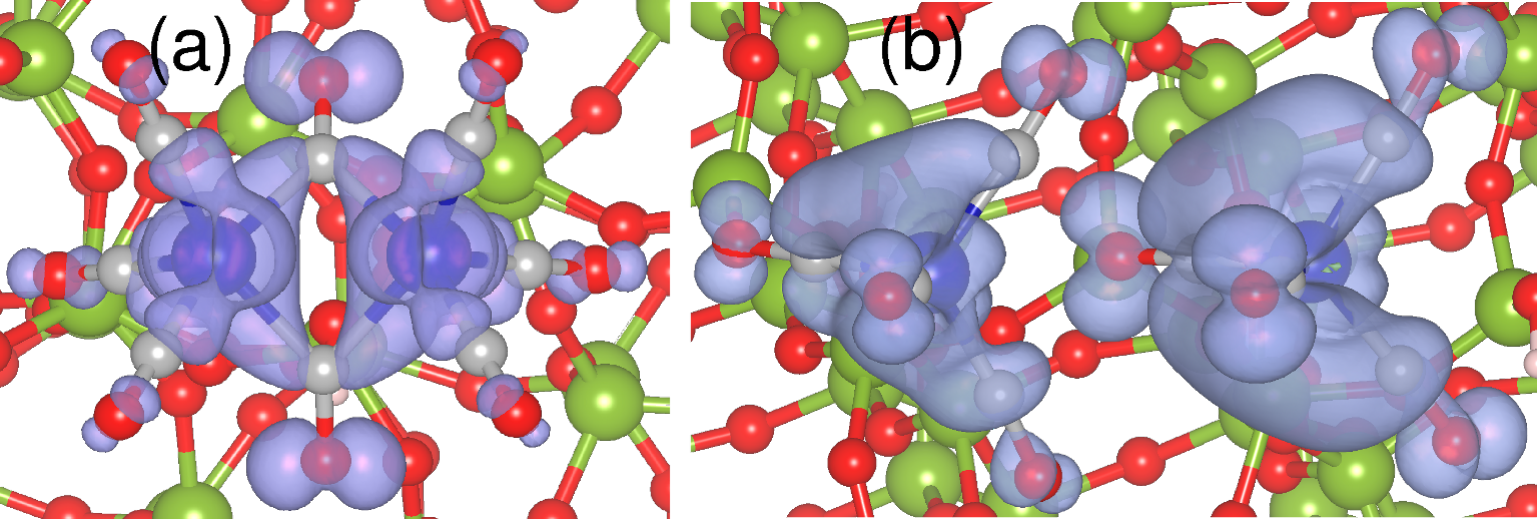
Band decomposed charge density for the valence band maximum for Co_2_(CO)_8_ on the (a) FOH-SiO_2_ and (b) POH-SiO_2_ surfaces.

The above results are in agreement with our experimental observations that the precursor molecules dissociate much more easily on the pretreated surfaces, as discussed in the previous section. In earlier experiments it was found that the decomposition of Co_2_(CO)_8_ depends on the different number of surface hydroxyls on the SiO_2_ substrates [23,60]. Although our calculations confirm that the molecule behaves differently on FOH-SiO_2_ and POH-SiO_2_ surfaces, we would like to note that the dissociation also depends on the orientation of the molecules. For example, on the POH-SiO_2_ surface the dissociation is observed only in two cases, i.e, when Co_2_(CO)_8_ is oriented in such a way that it bonds through one terminal and one bridging ligand, and when it is bonded through two terminal ligands. In particular, we did not observe any dissociation in C1, which has been believed to be the prominent mode of interaction with the weakly acidic hydroxylated surfaces in previous studies [59,61]. However, our results have been obtained by relaxing the initially prepared configurations to *T* = 0 directly; further studies on the thermal stability of Co_2_(CO)_8_ on POH-SiO_2_ in C1, C2, and C5 configurations are required. Moreover, the calculated charge density for the highest occupied valence band of Co_2_(CO)_8_ adsorbed on FOH-SiO_2_ and POH-SiO_2_ confirms that the molecule retains its character on FOH-SiO_2_ (compare Figure 5c and Figure 8a), but is strongly altered on the POH-SiO_2_ surfaces (compare Figure 5c and Figure 8b).

### Discussion on the dissociation and autocatalytic deposition of Co_2_(CO)_8_ precursor on SiO_2_ surface

In view of the results presented in the previous section, we will discuss here the possible reasons for dissociation and autocatalytic deposition of Co_2_(CO)_8_ molecules on SiO_2_ surfaces. The bridging CO ligands of Co_2_(CO)_8_ possess, in the free molecule, relatively higher electron density compared to the terminal ligands (Table 2, second column) and therefore are expected to be the ligands that preferentially interact with the dehydroxylated Si sites on the POH-SiO_2_ surface. Our results illustrate that, while the adsorption through the bridging ligands is essential, the terminal ligands are also involved in bonding to both FOH-SiO_2_ and POH-SiO_2_ surfaces. Let us focus now on the dissociation process of Co_2_(CO)_8_ on the POH-SiO_2_ surface, resulting in the formation of Co(CO)_4_ subcarbonyl motifs. The interaction between the CO ligands of the molecule precursor and the dehydroxylated Si sites of the surface alters the electronic distribution on the precursor molecule as well as its geometry. The changes in the electronic distribution are verified by the computed Bader charges on the CO ligands (Table 2, second and fourth columns) as well as on the Co atoms, in which the charge changes from +0.74 electrons in the free molecule to +0.54 electrons upon adsorption. This electronic change is accompanied by a structural change. The bond between C and O in the bridging CO ligand weakens (it elongates from 1.16 Å in the free molecule to 1.25 Å in the adsorbate) and the Co–C bond strengthens (it shortens from 1.95 Å in the free molecule to 1.66 Å in the adsorbate). Further, the bond angle (Co–C=O) in the bridging ligands changes from 140 to 174°. In addition, the surface Si atoms acquire a more positive character (the charge increases from +2.35 to +3.2 electrons) illustrating that this transfer of nearly one electron each from the two terminal Si sites on to the Co_2_(CO)_8_ molecule plays a crucial role in the fragmentation process. This accumulation of additional electron density on the individual Co atoms should weaken the bonding between the two Co atoms in the precursor. These effects, such as the strong bond (Si–CO) formation followed by the electronic redistribution in the precursor molecule, are further assisted by the interaction of the terminal carbonyl (see C4 in Figure 6) with the surface sites, which cleaves the molecules into two Co(CO)_4_ fragments.

**Table 2:** Calculated Bader charges for Co_2_(CO)_8_ in units of electrons in the gas phase and for the adsorbate on SiO_2_ surfaces. The numbers in parenthesis identify the CO ligand as shown in Figure 5a and Figure 7. Values indicated by an asterisk correspond to the total charge of the Co(CO)_4_ fragments

case	gas-phase	FOH-SiO_2_	POH-SiO_2_

CO(1)	−0.29	−0.24	−0.78
CO(2)	−0.29	−0.26	−0.24
CO(3)	−0.14	−0.09	−0.16
CO(4)	−0.14	−0.11	−0.22
CO(5)	−0.15	−0.06	−0.21
CO(6)	−0.15	−0.10	−0.23
CO(7)	−0.15	−0.12	−0.76
CO(8)	−0.16	−0.10	–0.15
Co1	+0.74	+0.55	+0.54
Co2	+0.74	+0.55	+0.54
total	+0.01	+0.02	(−0.83/−0.84)^*^

In contrast, Co_2_(CO)_8_ binds weakly on the FOH-SiO_2_ surface compared to POH-SiO_2_ (see Table 1) and it retains a similar character to that of the free molecule (compare Figure 5a and Figure 8a). Analysis of the charges on the CO ligands (Table 2, second and third columns) confirm this observation. Nevertheless, the formation of hydrogen bonds with surface hydroxyls leads to some charge redistribution within the adsorbed molecule, resulting in a reduction of positive charge from +0.74 to +0.55 on Co. Also, we find minimal differences in structural parameters (on the order of 0.01 Å).

The above observations illustrate the fact that the weak interaction between molecule and surface will not cause dissociation of the precursor. However, we would like to note that we have observed spontaneous dissociation of Co_2_(CO)_8_ in our experiments after extended exposure of the precursor flux (30 min or more). The spontaneous dissociation under long-time exposure is likely just a sign of the instability of the molecule which dissociates under CO release over the intermediate Co_4_(CO)_12_ at 52 °C. At lower temperature some degree of this dissociation will already be observable, in particular if there is no stabilizing CO atmosphere, such as is the case in a SEM vacuum chamber. (Moreover, the reduced neighbor coordination of the adsorbed molecules as compared to the bulk solid may speed up the dissociation process.)

In summary, our calculations confirm that Co_2_(CO)_8_ decomposes upon its interaction with POH-SiO_2_ surfaces, illustrating what may be the first step occurring in this deposition process. Furthermore, Co_2_(CO)_8_ molecules possess the capability to deposit autocatalytically as a result of spontaneous dissociation. At present it is unclear how to rationalize this autocatalysis, and a detailed study based on molecular dynamic simulations is in progress but beyond the scope of the present work. We expect that the total charge on the fragmented species of Co_2_(CO)_8_ is among the important factors that cause autocatalytic deposition. In our calculations, these fragments possess a net charge of −0.84 electrons. This charge is expected to play a similar role as the surface Si atoms on the POH-SiO_2_ surface, namely, it activates the approaching molecule and triggers the autocatalytic process. This indeed accounts for the fact that, in our experimental observations, the deposition occurs immediately on the pretreated surface, on which the fragments are formed as soon as the precursor flux is in contact with the POH-SiO_2_ surface, and with a slight delay on the FOH-SiO_2_ surface. However, this needs to be confirmed with theoretical simulations and remains as an open question that will be addressed in our future studies.

## Conclusion

We report here the deposition of Co from the precursor Co_2_(CO)_8_ on two different pretreated SiO_2_ surfaces, and our results provide an in-depth understanding of preliminary interactions and evidence for the spontaneous dissociation. Our observations suggest an activation of silica surfaces, which is also effective, although to a lesser degree, on Au layers. In view of the fact that no such spontaneous dissociation effects on Si substrates with a very thin native oxide layer have been reported in previous works [13,18], we are led to assume that this surface activation process depends on both a modified surface termination and trapped charges. Presently it is not clear whether the activation process observed on silica layers under ultrahigh vacuum conditions in conjunction with the precursor Fe(CO)_5_ [31] is also at work here. Further, we have also performed DFT calculations for this deposition process considering various slab settings, and we find that the extent of surface hydroxylation and the orientation of the precursor plays a vital role in the dissociation and the formation of the nanocomposites. The so-formed sub-carbonyl motifs during the FEBID process may be the true precursor for the Co-rich nanocomposite formation.
